# Lessons Learned for the Study of Childhood Asthma from the Centers for Children’s Environmental Health and Disease Prevention Research

**DOI:** 10.1289/ehp.7671

**Published:** 2005-06-24

**Authors:** Peyton A. Eggleston, Greg Diette, Michael Lipsett, Toby Lewis, Ira Tager, Rob McConnell, Elizabeth Chrischilles, Bruce Lanphear, Rachel Miller, Jerry Krishnan

**Affiliations:** 1 Johns Hopkins University School of Medicine, Baltimore, Maryland, USA; 2 University of California at San Francisco School of Medicine, San Francisco, California, USA; 3 University of Michigan School of Medicine, Ann Arbor, Michigan, USA; 4 University of California at Berkeley School of Public Health, Berkeley, California, USA; 5 University of Southern California School of Medicine, Los Angeles, California, USA; 6 University of Iowa College of Public Health, Ames, Iowa, USA; 7 University of Cincinnati School of Medicine, Cincinnati, Ohio, USA; 8 Columbia University College of Physicians and Surgeons, New York, New York, USA

**Keywords:** asthma, children, Children’s Centers, environmental health, National Children’s Study, pregnancy

## Abstract

The National Children’s Study will address, among other illnesses, the environmental causes of both incident asthma and exacerbations of asthma in children. Seven of the Centers for Children’s Environmental Health and Disease Prevention Research (Children’s Centers), funded by the National Institute of Environmental Health Sciences and the U.S. Environmental Protection Agency, conducted studies relating to asthma. The design of these studies was diverse and included cohorts, longitudinal studies of older children, and intervention trials involving asthmatic children. In addition to the general lessons provided regarding the conduct of clinical studies in both urban and rural populations, these studies provide important lessons regarding the successful conduct of community research addressing asthma. They demonstrate that it is necessary and feasible to conduct repeated evaluation of environmental exposures in the home to address environmental exposures relevant to asthma. The time and staff required were usually underestimated by the investigators, but through resourceful efforts, the studies were completed with a remarkably high completion rate. The definition of asthma and assessment of disease severity proved to be complex and required a combination of questionnaires, pulmonary function tests, and biologic samples for markers of immune response and disease activity. The definition of asthma was particularly problematic in younger children, who may exhibit typical asthma symptoms sporadically with respiratory infections without developing chronic asthma. Medications confounded the definition of asthma disease activity, and must be repeatedly and systematically estimated. Despite these many challenges, the Children’s Centers successfully conducted long-term studies of asthma.

In this article we outline information from the Centers for Children’s Environmental Health and Disease Prevention Research (Children’s Centers) that have conducted studies related to asthma. We do not include information on sampling strategies because the goal of the monograph is to inform the National Children’s Study. Rather, we focus on major issues related to the identification of asthma, asthma-related symptoms and end points, relevant exposures, biologic markers, and follow-up requirements.

Studies of asthmatic children and their homes were conducted at seven Children’s Centers. The protocols for these studies are summarized in Table 1, and further information is provided in the introduction to this mini-monograph (Kimmel et al. 2005). The birth cohort studies conducted at two Children’s Centers are described more completely by Eskenazi et al. (2005); they are included here because both Children’s Centers address respiratory cross-sectional data and outcomes. In addition, three Children’s Centers conducted cohort studies with older children with established asthma, and the study at Johns Hopkins University (JHU) included a nonasthmatic control group as a comparator in initial observations. Four Children’s Centers conducted intervention trials, with diverse designs ranging from a randomized primary prevention trial in school-age children to formal randomized controlled clinical trials.

## Recruitment of Participants

The studies that recruited children with asthma had key similarities (Table 1). Children were recruited whose parents reported a history of doctor-diagnosed asthma. Other children were recruited as controls. There were few exclusion criteria in most studies except for other respiratory diseases. Because all participants in these studies were minors, the investigators had to obtain informed consent from a parent or other guardian. When recruiting children > 7 years of age, assent was obtained from the child in addition to the consent obtained from a parent or guardian. In the two Children’s Centers that conducted birth cohort studies, mothers consented to obtain information regarding their own and their child’s medical and exposure histories (Eskenazi et al. 2005). In certain cases, the study setting included prespecified areas of a city bounded by ZIP codes or prespecified counties. In other cases, the study setting was defined by participants seeking care at a specific clinic or medical center regardless of where they lived.

Although three studies used local schools (University of Iowa, JHU intervention study, University of Michigan), most studies identified the sample from a health care setting such as health plans, emergency departments, and physician offices. In schools, the protocol was approved both by an institutional review board (IRB) and by a school system IRB. Informed consent was mailed to the parents to be signed, and followed by a mailed questionnaire in the case of Iowa and Michigan; in the JHU intervention study, the mailed consent was followed by a visit from a recruiter. For other studies, informing and recruiting potential participants included both passive and active methods. The passive approaches included posting study information publicly and providing information when a potential participant’s parent or guardian called. These were generally less efficient in generating interested families to participate and generally were considered a supplement to active methods. For the studies of children, most active recruitment consisted of generating patient lists from computerized databases of children who met entry criteria. Study information was then mailed directly to participants, and study staff telephoned sometime later to determine the family’s interest, to confirm eligibility, and to initiate the informed consent process.

## Barriers to Recruitment

There were several barriers to recruitment of children to the studies. First among these was establishing the essential entry criterion for a diagnosis of asthma. This was a greater issue for those studies recruiting younger children (JHU longitudinal cohort study, Cincinnati) than in those focused on older children [JHU Intervention trial, University of Southern California (USC), Michigan, Iowa]. Older children were generally identified by a diagnosis being made by a physician and logged into the computerized database from which recruitment began. In some cases, children with typical asthma were entered into databases with related diagnoses such as recurrent bronchitis or reactive airway disease, which have slightly different codes in the International Classification of Diseases, 9th Revision [World Health Organization (WHO) 1978]. Study staff confirmed the diagnosis with screening questionnaires defining appropriate symptoms or with lung function tests. Younger children, who may have had asthmatic respiratory symptoms only during respiratory infections, were more difficult to qualify because they had less specific answers to respiratory questionnaires and could not perform lung function tests. In a birth cohort study, early wheeze may be recorded, with persistent wheeze or asthma being defined as an outcome later in life. However, entry criteria for the longitudinal cohort studies that recruited younger children used the same definition used with older children: doctor-diagnosed asthma. In a birth cohort study, cough or wheeze may be recorded as an outcome, with persistent wheeze or definable asthma used as an outcome later in life.

An important barrier was introduced by the need to preserve patient autonomy and privacy. IRBs have long required investigators to accommodate patients listed on health care databases who do not want to be contacted to participate in research studies. This has usually been accomplished by mailing an invitation letter containing a preaddressed and stamped postcard that families can return requesting that they not be called. In general, this has proven to be a minor barrier, excluding < 5% of identified families. The recently issued Health Insurance Portability and Accountability Act (HIPAA) regulations have introduced additional barriers by requiring that families give permission to use their health information even at preliminary stages of research (e.g., investigators having access to the health care database to obtain lists of children with a diagnosis of asthma). For example, HIPAA makes it illegal to create lists of persons with a specific diagnosis to whom to send an introductory letter and makes the approach used by most of these studies illegal. Various solutions have been developed, including having patients sign general waivers of HIPAA privacy rights as they enroll in a health care program or having their health care provider recommend them to the interviewer at the time of a health care encounter. Other approaches have included asking asthmatic patients or their parents who participate in health fairs, surveys, self-management programs, or other patient care activities to indicate in writing their willingness to participate in future research studies and creating a list of those who have so indicated. IRBs may approve a HIPAA waiver, but the investigators must justify why a waiver is necessary—that is, that the data being gathered are not sensitive, are not able to be linked with individual identifiers, or both are noninvasive and cannot be collected in another way. This requirement also involves clinic personnel, who are much less effective as recruiters that are trained, motivated study staff. Recruitment from school directories avoids these problems because health data are not used to identify potential subjects. One study that recruited from schools did so by conducting a mailed asthma screening survey of 10 school districts (87% response rate) to identify children with asthma (Chrischilles et al. 2004). Another reported a 78% response rate from 9,437 mailings (Lewis et al. 2004).

The two birth cohort studies (University of California at Berkeley, Columbia University) recruited pregnant mothers and thus faced the issue of participant identification to a smaller extent. At the time the studies were initiated, the HIPAA regulations had not been activated, so they did not face this issue. Were the studies to be conducted now, recruitment of identified pregnant mothers (with or without asthma) would still have to comply with the HIPAA requirements and, indeed, do so in the continued surveillance of the children.

A final barrier when dealing with families with lower socioeconomic status is obtaining a reliable means to contact persons on a list. Families move frequently (29% moved at least once during a 1-year observation) (Swartz et al. 2004), so the address and telephone numbers in a database may not be current. Additionally, as many as 25% of inner-city families may not have telephones (Wissow et al. 1988), and 52% may change their telephone number at least once during a year of follow-up (Swartz et al. 2004). This not only makes initial contact difficult but also interferes with follow-up.

## Retention

As shown in Table 1, retention rates were generally > 80% in those studies that involved follow-up. A number of barriers had to be overcome to achieve these high rates, including frequent telephone number changes, residential address changes, and general reluctance in making follow-up appointments. The usual solutions to the lack of telephones or frequent changes of telephones and addresses are to maintain frequent contacts with participants (generally every 1–2 months), to ask families for secondary (and tertiary) alternative contact information, or to provide incentives to notify the study personnel in the event of a change in address or telephone number (Mitchell et al. 1997). Most study staff members were able to conduct home visits even when telephone contact strategies had proven unsuccessful. However, families who moved without providing new contact information interfered with this strategy as well as with follow-up home inspections that were part of several protocols. For some Children’s Centers, missed appointments were important barriers to retention. Reasons for missed appointments included inclement weather and illness among family members needed to help transport the child, but in many cases no explanation was provided. Some studies categorized repeated “no-shows” as having passively withdrawn from the study. This practical measure was necessary to preserve study resources. Children’s Centers that conducted the research in the homes of subjects (i.e., did not require participants to come to the study center) also faced appointment cancellations.

The Children’s Centers employed multiple strategies to maintain contact with participants and to encourage their continued participation. Commonly used incentives included cash, gift certificates, toys for the children, food stamps, and infant car seats. A wide variety of gifts were provided, including T-shirts, tote bags, hats, key chains, stuffed animals, games, and musical instruments. These incentives were provided either at follow-up visits or on successful completion of the study. Additional incentives included health-related devices such as peak flow meters, spacers for metered dose inhalers, and allergen-proof mattress and pillow covers. One Children’s Center provided up to $200 as reimbursement for electricity costs incurred during the study. The Columbia Children’s Center, faced with participants who moved to a new location (e.g., Florida), paid for air travel to do follow-up assessments, and in one case a research worker has flown to the Dominican Republic to obtain follow-up data. The Berkeley Children’s Center created a movable laboratory in a recreation vehicle and went on the road once a year to other areas in California to include participants who moved. Another common incentive was to provide reimbursement for travel expenses related to study participation.

In some cases, the incentives had no monetary value but focused on information provided to the family. In the JHU cohort study, participants learned about home environmental measurements, including allergens and pollutants; many of these families indicated that this was the principal incentive that attracted them to the study. All investigators felt that the most important factor in participant retention was the Children’s Centers’ staffs. Retention was highest when the staff members were able to create an empathetic bond with the participating families, who felt that the staff would try to help them not only with their child’s illness but also with other difficulties associated with their social and economic circumstances. Families frequently sought help with problems such as transportation, referrals, and problems of daily living and valued the “ear” provided by the staff regardless of whether the staff member could actually help with the issue.

## Environmental Data

As shown in Table 2, all the Children’s Centers employed extensive questionnaires, collecting demographic, social, medical, and environmental exposure data similar to those collected in other studies. These data were collected repeatedly during longitudinal follow-up, although generally in abbreviated questionnaires.

Most Children’s Centers conducted ambient air sampling at one or more sites (Table 3). In addition, all Children’s Centers conducted home visits and in many cases conducted visits repeatedly (see Table 4). At these visits, the participants’ homes were formally inspected with checklists and families provided additional information through questionnaires. In most cases, settled dust samples were collected and were assayed for indoor allergens; concentrations of endotoxin and pesticides were also measured. Most Children’s Centers conducted repeated measures of environmental exposures, including inspection, settled dust sampling, and air sampling; completion rates for these evaluations ranged from 73 to 91%. The data were generally used to describe exposure at the times indicated and was compared with asthma morbidity at these times. Because the data were collected as several time points, time series analyses were conducted.

## Biologic Samples

The number of studies that collected blood samples from children was remarkable (Table 5). These samples were generally used for radioallergosorbent tests (RAST) for specific immunoglobulin E (IgE) antibody to supplement or replace skin tests. Prick skin tests and RAST have been shown to correlate well for sensitivity to inhalant allergens (Wood et al. 1999) and food allergens (Sampson and Albergo 1984). When blood is saved for RAST, the investigator has the advantage of being able to test for additional sensitivities that were not considered at the time the study was conducted. The specific sensitivities determined in this manner were essential both to determine that a child was atopic and to detect specific IgE to the indoor allergens measured in home visits. Atopy, defined as a genetic predisposition to produce long-lived IgE antibody to environmental allergens in association with a constellation of chronic diseases including food allergy, eczema, allergic rhinitis, and asthma, is the most important risk factor for both incident asthma and asthma severity. The combination of specific IgE to environmental allergens and the presence of high concentrations of these allergens in the child’s home is the strongest known risk factor for asthma severity and morbidity (Rosenstreich et al. 1997).

In addition to blood samples, prick–puncture skin testing was usually done, and samples of meconium, urine, saliva, and hair were collected, usually to detect exposure to environmental tobacco smoke (ETS) and pesticides. What is most remarkable is that biologic samples were collected from children in most Children’s Centers on multiple occasions and that these collections did not have noticeable impact on participant retention rates (Table 1).

## Asthma Disease Activity

All of these studies included questions regarding asthma disease activity as shown in Table 6. Assessing disease morbidity in epidemiologic studies of children with asthma involves the creation of a composite of symptoms, health care, and medication use from questionnaires, together with pulmonary function tests in older children (Kattan et al. 1997). Although there is a general consensus about asthma-associated symptoms, there is less agreement with regard to specific questions that provide the most appropriate assessment of these symptoms. Major symptoms include cough, wheeze, chest tightness, and dyspnea, but these are quite variable day to day and depend on exposures to specific stimuli or “triggers” such as respiratory viruses, allergens, or irritants such as ETS and air pollutants. It is now generally accepted that symptoms should be assessed over 2-week intervals and that questions be framed in terms of the number of days during which these symptoms are experienced rather than frequency per day or symptom intensity. The two most widely used questionnaires were developed by the American Thoracic Society (ATS) (Ferris 1978) and the Children’s Health Survey for Asthma (CHSA) (Asmussen et al. 1999).

Several scales have been created to synthesize the symptoms into a description of disease activity. The most commonly accepted comes from the National Institute of Health (NIH)’s National Asthma Education and Prevention Program (NAEPP) (National Heart, Lung, and Blood Institute 1997). This scale, summarized in Table 7, was originally intended to categorize severity in untreated asthmatics in clinical settings but has been used in epidemiologic studies, as well (Diette et al. 2001). More recently, as the use of daily medication for asthma has become more common, the NAEPP scale has been felt to more appropriately describe disease control rather than severity.

Some of the Children’s Centers included questions from the CHSA (Asmussen et al. 1999) or from the pediatric version of the ATS questionnaire. The JHU intervention study used the ATS questions as they had been adapted for the Children’s Asthma Management Program study (Szefler et al. 2000). The difference between these questionnaires is that the CHSA uses a Likert scale to define symptom frequency in the preceding 2 weeks, whereas the other questionnaires ask how many days the child had experienced individual symptoms; the reliability of the CHSA is better documented than is the case with other questionnaires. Because of the diurnal variation in asthma disease activity, symptoms occurring at night and during the day are always considered separately. Symptoms occurring with exercise or on days that the child does not have an upper respiratory tract infection are also recorded separately.

Other aspects of asthma morbidity also recorded in the Children’s Centers’ investigations included interference with a child’s activity (exercise, play, school, sleep), interference with parents’ activities, and acute events. The latter were generally defined as those requiring systemic corticosteroid use, unscheduled physician visits, visits to emergency rooms, or hospitalization. Because these were uncommon events, the recall period was generally longer, that is, 2 months or longer, in almost all Children’s Centers.

In the early 1990s, an international group created five core questions regarding childhood asthma to facilitate an international comparison of varying prevalence rates or severity indices. This scale from the International Study of Asthma and Allergy in Childhood (ISAAC) (Asher et al. 1995) has widespread support among epidemiologists. The ISAAC core asthma questions were commonly included in the asthma-specific questionnaires used in the Children’s Centers’ studies.

Juniper et al. (1996, 2000) developed a series of questions to assess the effect of symptoms on a child’s quality of life. These provide a composite assessment of disease activity and tend to correlate better with daily lung function measures than do symptom questionnaires. In addition, they have the advantage of providing a single summary number that has proven to be sensitive to change across time, an extremely valuable property in longitudinal cohort studies and interventions.

Pulmonary function tests are important measures of disease activity but correlate modestly with reported symptoms or other measures of morbidity. Daily measures usually are limited to peak expiratory flow rate (PEFR), although forced expiratory volume in 1 sec (FEV_1_) may also be measured by portable spirometers. Children, parents, and staff must be trained in the use of these devices; most preschool children given training can perform accurate PEFR measures. With proper coaching, 83% of children 3–6 years of age can complete technically acceptable and reproducible maneuvers (Eigen et al. 2001); the Children’s Center’s experience with technically acceptable data ranged from 52 to 99%.

Medication use both modifies symptoms and provides an independent measure of disease morbidity. Medications that are taken to reverse symptoms of obstruction [“relievers” is the term used in the NIH consensus guidelines (National Heart, Lung, and Blood Institute 1997)] are recorded as equivalent to symptoms during a day. Medications that are taken daily [“controllers” in the NIH consensus guidelines (National Heart, Lung, and Blood Institute 1997)] may modify symptoms, as may medications taken before exercise or other stimuli to prevent attacks. In this setting, it is appropriate to talk about disease “control” rather than “morbidity.” Validated scales have been published to describe disease control in adults (Nathan et al. 2004).

Diaries can potentially give more accurate records of disease activity, but retrieval and consistency are problematic. Diaries and periodic questionnaires generally correlate well (Gold et al. 1989), and the studies conducted in the Children’s Centers used repeated questionnaires to avoid the logistical problems of diary retrieval and data verification.

## Interventions

Five of the Children’s Centers conducted interventions to test the efficacy of environmental control measures on improving indoor environmental exposures and asthma-related health. Strategies employed are summarized in Table 8. Although all Children’s Centers emphasized an environmental education program for families, they varied in the breadth and intensity of other components of the intervention programs. Some Children’s Centers focused on strategies targeting a few key triggers, whereas others chose a more comprehensive approach. In addition, some Children’s Centers chose to supplement home environmental strategies with education for families on asthma management, or education targeting physicians treating the asthmatic children. The relative benefits and challenges of these various strategies remain the subject of intense investigation.

## Local Variations

Although childhood asthma is an issue of national and international significance, it is important to remember that it occurs within a local context. Cultural, social, and linguistic factors vary by location, as do systems of health care and community resources. Sources of environmental exposure, housing stock, and population behavior patterns relative to the exposure may vary tremendously. In addition, historical relationships between academia and local communities may range from strained to quite cooperative. Each of these factors influences the way asthma studies and interventions can be practically implemented in any given location. Some of the variation seen in study design between Children’s Centers is a direct result of variation in local priorities, circumstances, and resource constraints. What may work in one setting may not be feasible or relevant in another. Each of the Children’s Centers described here used a community-based participatory research approach (Israel et al. 2005) in which community partners contributed significantly to the research process. The Children’s Centers universally report that the involvement by community members enhanced their ability to accomplish their research goals. Multicenter studies, such as the proposed National Children’s Study, have an additional challenge of balancing the need for protocol uniformity across sites with the very real need to adjust to local contextual issues.

## Conclusions: Lessons Learned

The experience gained in the studies conducted by the National Institute of Environmental Health Sciences/U.S. Environmental Protection Agency Children’s Centers for Children’s Environmental Health and Disease Prevention provide important lessons for the National Children’s Study. The lessons that specifically relate to asthma can be summarized as follows:

- Asthma identification requires a combination of questionnaire and physiologic measures. Many validated questionnaires are available to record asthma symptoms in children; these differ in the terms used to describe symptoms and in the period of recall. To allow comparison with previously reported data and to allow data regarding children in the United States to be compared with those of children in other countries, it is advisable to include questions from the ISAAC, the ATS, and the CHSA in study questionnaires. In addition to historical information, objective measures such as spirometry, eosinophil counts in peripheral blood or secretions, or measures of specific IgE antibody are usually included in definitions of asthma.
- Identification of asthma in preschool-age children is problematic. Many children wheeze or cough with respiratory infections and never wheeze when they are older. These episodes are not considered to be asthmatic, and current methods only modestly predict which infants with wheezing will later develop asthma. For this reason, it is appropriate to classify these episodes as recurrent wheezing illness rather than asthma and to reserve the definition of asthma for older children with more persistent symptoms.
- Medication confounds the assessment of asthma symptoms and classification of disease severity. Short-acting β-adrenergic agonists (SABAs) will predictably improve acute asthma symptoms. In questionnaire histories, it is appropriate to equate the use of these medications with episodes of asthma; the fact that the SABAs are often used to prevent symptoms introduces uncertainty into this statement. Daily controller medications are used preventively and are currently used in more severe cases, so the use of these medications usually indicates more severe disease; however, the inconsistency with which these medications are prescribed lends considerable uncertainty to this statement.
- Recruitment and data collection in health care settings require dedicated study staff. The Children’s Centers found that health care personnel in clinical settings could not be relied on either to recruit children into the studies or to collect outcome data. This added to the cost of recruiting in clinical settings and, in addition, added to the complexity of collecting health information from patients whose privacy was protected by HIPAA regulations. When accessed in a manner consistent with HIPAA guidelines, medical records can provide useful supplemental information, but in most clinics and hospitals the lack of standardized records makes this information less useful.
- There are important longitudinal data to be gained from cohorts of older children. Longitudinal studies provide essential data regarding the sequence of exposure to environmental agents and incidence cases of asthma. Similarly, these studies provide important exposure response data with regard to the sequence of asthma episodes and environmental exposures in symptomatic asthma. Asthma is characteristically variable, so repeated measures are important. The frequency of asthmatic symptom recall and the variability of important environmental stimuli dictate how frequently these data must be recorded. For example, daily symptoms are best explained by environmental measures made during the same days. However, environmental samples have traditionally been collected for several (or many) days to accommodate analytic sensitivity, but paradoxically, it is difficult to attribute symptoms reported on some but not all days during which the measures were made. It also follows that sporadic events, such as hospitalizations, are difficult to associate with measures averaged over several months.

## Figures and Tables

**Table 1:** Overview of asthma-related studies and intervention.

	Berkeley	Columbia	USC	JHU	Michigan	CCH	USC	JHU	Iowa	Michigan
Study design	BC	BC	LC	LC	LC	RCT	RCT	RCT	IT	RCT
Sample size (*n*)	601^a^	861^a^	11,841	150 case, 150 control	298^b^	225	202	100	189	298^b^
Outcomes	Respiratory symptoms, atopy, medications, health care use	Respiratory symptoms, PFT, medications, health care use	Asthma symptoms	Asthma symptoms, medications, PEFR, health care use	Asthma symptoms, PEFR	Asthma symptoms, medications, health care use, child behavior	Asthma symptoms, medications, health care use	Asthma symptoms, medications, health care use, FEV_1_	Asthma symptoms, health care use, management behaviors	Asthma symptoms, medications, health care use, PEFR, FEV_1_
Exposures	Pesticides, ETS, dust allergens (home and ambient), dust endotoxin, social stressors	PM, DEP, PAH, ETS, pesticides, dust, allergens, social stressors	Air pollution, dust allergens, social stressors	PM, NO_2_, O_3_ (home and ambient), dust allergens, ETS	PM (ambient, home, personal), O_3_, ETS	ETS, dust allergens, social stressors	Dust allergens, social stressors	PM, NO_2_, O_3_ (home and ambient), dust allergens, ETS	Dust allergens, dust endotoxin, ETS	Dust allergens, endotoxin, ETS, social stressors
Retention (%)^c^	86	90	78	83	67	96	77	93	76	77

Abbreviations: BC, birth cohort; CCH, Cincinnati Children’s Hospital; DEP, diesel exhaust particles; IT, intervention trial; LC, longitudinal cohort; NO_2_, nitrogen dioxide; O_3_, ozone; PAH, polycyclic aromatic hydrocarbon; PFT, pulmonary function tests; PM, particulate matter; RCT, randomized controlled clinical trial.

a Pregnant women.

b The same children were studied in the intervention trial and the longitudinal cohort in Michigan. The two studies were conducted simultaneously, with the longitudinal observational study extending follow-up after completion of the intervention.

c Time interval for retention varies across studies.

**Table 2:** Questionnaire information.

Characteristic	Berkeley	Columbia	USC: LC	JHU: LC	Michigan: LC	CCH	USC: IT	JHU: IT	Iowa	Michigan: IT
Collection schedule	Pregnancy, birth, 6,12, 24, 42 months of age	Pregnancy, every 3 months until age 24 months; every 6 months until 60 months of age	Baseline, yearly	Baseline, 3, 6 months	Baseline, 3, 6, 9, 12, 15, 18, 21, 24, 27, 30 months	Baseline, day 2, 3, 6, 9, 12, 12.5 months	Baseline, 4, 8, 12, 16, 20 months	Baseline, 3, 6, 9, 12 months	Baseline, 12 months	Baseline, 12 months
Demographics	X	X	X	X	X	X	X	X	X	X
Occupation	X	X		X		X	X		X	
Housing characteristics	X	X	X	X	X	X	X	X	X	X
Pesticide exposure	X	X	X						X	X
Allergen exposure	X	X	X	X	X	X	X	X	X	X
Cleaning habits	X	X				X	X	X	X	X
Social support	X	X	X	X		X	X	X	X	X
Maternal depression	X	X		X		X		X		X
Child diet	X	X	X	X				X		
Respiratory symptoms	X	X	X	X	X	X	X	X	X	X
Medication use	X	X	X	X	X	X	X	X	X	X
Home remedies	X	X							X	X
Smoking	X	X	X		X	X	X			X
Medical history	X	X	X	X	X	X	X	X	X	X
Household income	X	X	X	X	X	X	X	X	X	X
Pets	X	X	X	X	X	X	X	X	X	X
Child care	X	X		X			X	X	X	

Abbreviations: BC, birth cohort; CCH, Cincinnati Children’s Hospital; IT, intervention trial; LC, longitudinal cohort.

**Table 3:** Ambient air sampling.

Sample	Berkeley	Columbia	USC: LC	JHU: LC	Michigan: LC	CCH	JHU: IT
Collection schedule	Pregnancy, 6, 12 months and at community site	Personal air during pregnancy, ambient and indoor at 12 months	Continuous at community site	Baseline, 6, 12 months	Baseline, 3, 6, 9, 12, 15, 18, 21, 24, 27, 30 months	6, 12 months	Baseline, 6, 12 months
PM_10_			X	X	X		X
PM_2.5_		X	X	X	X		X
NO_2_			X	X			X
Ozone			X	X	X		X
Mold/pollen	X	X					
Air nicotine						X	

Abbreviations: BC, birth cohort; CCH, Cincinnati Children’s Hospital; IT, intervention trial; LC, longitudinal cohort; PM, particulate matter; PM_2.5_, PM < 2.5 μm in diameter; PM_10_, PM < 10 μm in diameter. Samples were not collected in USC: IT, Iowa, and Michigan: IT.

**Table 4:** Home evaluation.

Characteristic	Berkeley	Columbia	USC: LC	JHU: LC	Michigan: LC	CCH	USC: IT	JHU: IT	Iowa	Michigan: IT
Collection schedule	Pregnancy, 6, 12, 24, 42 months	Pregnancy, 12, 24 months	For subsample, one time	Baseline, 3, 6 months	Baseline, 12, 24 months	Baseline, 6, 12 months	Baseline, 4, 8, 12, 16, 20 months	Baseline 6, 12 months	Baseline, 12 months	Baseline, 12 months
Age of house				X	X	X	X	X	X	X
Proximity traffic	X	X	X	X		X	X	X		
Proximity fields	X								X	
Flooring type, condition	X			X	X	X		X	X	X
Cockroaches	X	X		X	X	X	X	X	X	X
Rodents	X	X		X	X	X	X	X	X	X
Mold	X	X		X	X	X	X	X	X	X
Wall moisture	X	X		X	X	X	X	X	X	X
Peeling paint	X	X		X	X			X		X
Water damage	X	X		X	X	X	X	X	X	X
Pets	X	X		X	X	X	X	X	X	X
Pesticide use	X	X			X		X		X	X
Gas stove/heater	X	X		X	X	X	X	X	X	X
Cleanliness	X			X	X	X		X	X	X
House dust	X	X	X	X	X	X	X	X	X	X
Allergens	X	X	X	X	X	X	X	X	X	X
Pesticide	X	X					X		X	
Endotoxin	X	X	X		X		X			X
Home air	X^a^			X	X^b^	X		X		X
PM_10_				X	X^b^			X		
PM_2.5_		X		X	X^b^			X		
NO_2_			X	X				X		
Ozone				X				X		
Allergen		X		X				X		
Endotoxin				X				X		
Nicotine				X	X	X				X

Abbreviations: BC, birth cohort; CCH, Cincinnati Children’s Hospital; IT, intervention trial; LC, longitudinal cohort; PM, particulate matter; PM_2.5_, PM < 2.5 μm in diameter; PM_10_, PM < 10 μm in diameter.

a Mold spores documented.

b Evaluated at baseline and every 3 months.

**Table 5:** Biologic samples.

Sample	Berkeley	Columbia	JHU: LC	Michigan: LC	CCH	USC: IT	JHU: IT	Michigan: IT
Blood	Maternal pregnancy and delivery; cord blood; 12, 24 months	Cord blood, 24, 36, 60 months	Baseline		Baseline, 6, 12 months		Baseline, 12 months	
Urine	Maternal pregnancy and delivery; 6, 12, 24, 42 months	Maternal pregnancy, 36, 60 months			6 months		Baseline	
Meconium		X						
Saliva	42 months							
Hair					Baseline, 6, 12 months			
Skin tests		X	X	X	X	X	X	X
RAST		X	X		X	X		

Abbreviations: BC, birth cohort; CCH, Cincinnati Children’s Hospital; IT, intervention trial; LC, longitudinal cohort. Samples were not collected in USC: LC and Iowa.

**Table 6:** Asthma disease activity.

Characteristic	Berkeley	Columbia	USC-LC	JHU-LC	Michigan-LC	CCH	USC-IT	JHU-IT	Iowa	Michigan-IT
Day symptoms		X	X	X	X	X	X	X	X	X
Night symptoms		X	X	X	X	X	X	X	X	X
Exercise symptoms		X	X	X	X	X	X	X	X	X
Activity limited		X		X	X	X	X	X	X	X
ISAAC questions^a^	X	X	X	X			X	X	X	X
Quality of life^a^		X				X	X	X	X	X
Symptom medications	X	X	X	X	X	X	X	X	X	X
Control medications	X	X	X	X	X	X	X	X	X	X
Oral steroids	X	X	X	X	X	X	X	X	X	X
Recall period^b^	6–12 months	3–6 months	1 year		Daily	2 weeks, 3 months	8 weeks	2 weeks	2 weeks	2 weeks
ED visits	X	X	X	X	X	X	X	X	X	X
Hospitalization	X	X	X	X	X	X	X	X	X	X
Recall period^c^	6–12 months	3 months	1 year	3 months	Daily	3 months	4 months	3 months	2 months	3 months, 1 year
FEV_1_		X	X		X	X	X	X	X	X
Daily FEV_1_					X	X				X
Daily PEFR				X	X	X	X			X
Allergy history	X	X	X	X		X	X	X		X
Family history	X	X	X	X		X	X	X	X	

Abbreviations: BC, birth cohort; CCH, Cincinnati Children’s Hospital; ED, emergency department; IT, intervention trial; LC, longitudinal cohort.

a One-year recall period for ISAAC, 1-week recall period for quality-of-life questions.

b Recall period for symptoms, medications.

c Recall period for emergency department, hospitalizations.

**Table 7:** NAEPP asthma activity classification.

Classification	Days with symptoms^a^	Nights with symptoms^b^	PEFR, FEV_1_ (%)^c^
Mild intermittent	0–1	0–1	> 80
Mild persistent	> 2	2	> 80
Moderate persistent	Daily	Weekly	< 80
Severe persistent	Constant	> Weekly	< 60

a Days during previous 2 weeks.

b Nights during previous month.

c Measures as a percentage of normal predicted for age, sex, and race.

**Table 8:** Interventions.

	Cincinnati	USC	JHU	Iowa	Michigan
Pest control	X	X	X		X
Allergen-proof mattress covers	X		X		X
HEPA air cleaners			X		
Vacuum cleaners with HEPA filters					X
Cleaning supplies			X		X
Environmental education	X	X	X	X	X
Asthma management education			X	X	X^a^
Smoking cessation	X	X	X		X^a^
Physician education				X	
Social support and referrals					X

a Provided only in a limited fashion.

## References

[b1-ehp0113-001430] Asher MI, Anderson HR, Beasley R, Crane J, Martinez F, Mitchell EA (1995). International study of asthma and allergies in childhood (ISAAC): rationale and methods. Eur Respir J.

[b2-ehp0113-001430] Asmussen L Olson LM Grant EN Fagan J Weiss KB 1999 Reliability and Validity of the Children’s Health Survey for Asthma Pediatrics 104 e71 Available: http://www.pediatrics.org/cgi/content/full/104/6/e71 [accessed 24 January 2005]. 10586005 10.1542/peds.104.6.e71

[b3-ehp0113-001430] Chrischilles E, Ahrens R, Kuehl A, Kelly K, Thorne P, Burmeister L (2004). Asthma prevalence and morbidity among rural Iowa schoolchildren. J Allergy Clin Immunol.

[b4-ehp0113-001430] Diette GB, Skinner EA, Nguyen TT, Markson L, Clark BD, Wu AW (2001). Comparison of quality of care by specialist and generalist physicians as a usual source of asthma. Pediatrics.

[b5-ehp0113-001430] Eigen H, Bieler H, Grant D, Christoph K, Terrill D, Heilman DK (2001). Spirometric pulmonary function in healthy preschool children. Am J Resp Crit Care Med.

[b6-ehp0113-001430] Eskenazi B, Gladstone EA, Berkowitz GS, Drew CH, Faustman EM, Holland NT (2005). Methodologic and logistic issues in conducting longitudinal birth cohort studies: lessons learned from the Centers for Children’s Environmental Health and Disease Prevention Research. Environ Health Perspect.

[b7-ehp0113-001430] Ferris BG (1978). Epidemiology standardization project. II. Recommended respiratory disease questionnaires for use with adults and children in epidemiologic research. Am Rev Respir Dis.

[b8-ehp0113-001430] Gold DR, Weiss ST, Tager IB, Segal MR, Speizer FE (1989). Comparison of questionnaires and diary methods in acute respiratory illness surveillance. Am Rev Respir Dis.

[b9-ehp0113-001430] Israel BA, Parker EA, Rowe Z, Salvatore A, Minkler M, López J (2005). Community-based participatory research: lessons learned from the Centers for Children’s Environmental Health and Disease Prevention Research. Environ Health Perspect.

[b10-ehp0113-001430] Juniper EF, Guyatt GH, Feeny DH, Ferrie PJ, Griffith LE, Townsend M (1996). Measuring quality of life in children with asthma. Qual Life Res.

[b11-ehp0113-001430] Juniper EF, O’Byrne PM, Ferrrie PJ, King DR, Roberts JN (2000). Measuring asthma control: clinic questionnaire or daily diary. Am J Respir Crit Care Med.

[b12-ehp0113-001430] Kattan M, Mitchell H, Eggleston P, Gergen P, Crain E, Redline S (1997). Characteristics of inner-city children with asthma: the National Cooperative Inner-City Asthma Study. Pediatr Pulmonol.

[b13-ehp0113-001430] Kimmel CA, Collman GW, Fields N, Eskenazi B (2005). Lessons learned for the National Children’s Study from the National Institute of Environmental Health Sciences/U.S. Environmental Protection Agency Centers for Children’s Environmental Health and Disease Prevention Research. Environ Health Perspect.

[b14-ehp0113-001430] Lewis TC, Robins TG, Joseph CLM, Parker EA, Israel BA, Rowe Z (2004). Identification of gaps in the diagnosis and treatment of childhood asthma using a community-based participatory research approach. J Urban Health.

[b15-ehp0113-001430] Mitchell H, Senturia Y, Gergen P, Baker D, Joseph C, McNiff-Mortimer K (1997). Design and methods of the National Cooperative Inner-City Asthma Study. Pediatr Pulmonol.

[b16-ehp0113-001430] Nathan RA, Sorkness CA, Kosinski M, Schatz M, Li JT, Marcus P (2004). Development of the asthma control test: a survey of assessing asthma control. J Allergy Clin Immunol.

[b17-ehp0113-001430] National Heart, Lung, and Blood Institute 1997. National Asthma Education and Prevention Program. Expert Panel Report 2: Guidelines for the Diagnosis and Management of Asthma. NIH Publication 97-4051. Bethesda, MD:National Heart, Lung, and Blood Institute.

[b18-ehp0113-001430] Rosenstreich DL, Eggleston PA, Kattan M, Baker D, Slavin RG, Gergen P, for the National Cooperative Inner City Asthma Study (1997). The role of cockroach allergy and exposure to cockroach allergen in causing morbidity among inner-city children with asthma. N Engl J Med.

[b19-ehp0113-001430] Sampson HA, Albergo R (1984). Comparison of results of skin tests, RAST and double-blind, placebo-controlled food challenges in children with atopic dermatitis. J Allergy Clin Immunol.

[b20-ehp0113-001430] Swartz LJ, Callahan KA, Butz AM, Rand CS, Kanchanaraksa S, Diette GB (2004). Methods and issues in conducting a community-based environmental randomized trial. Environ Res.

[b21-ehp0113-001430] Szefler SJ, Weiss S, Tonascia J, for the Childhood Asthma Management Program Research Group (2000). Long-term effects of budesonide or nedocromil in children with asthma. N Engl J Med.

[b22-ehp0113-001430] WHO 1978. International Classification of Diseases, 9th Revision. Geneva:World Health Organization. 10237209

[b23-ehp0113-001430] Wissow LS, Warshow, Box J, Baker D (1988). Case management and quality assurance of improved care of inner-city children with asthma. Am J Dis Child.

[b24-ehp0113-001430] Wood RA, Phipatanakul W, Hamilton RG, Eggleston PA (1999). A comparison of prick skin tests, intradermal skin tests, and RASTs in the diagnosis of cat allergy. J Allergy Clin Immunol.

